# Uncovering sarcopenia and frailty in older adults by using muscle ultrasound—A narrative review

**DOI:** 10.3389/fmed.2024.1333205

**Published:** 2024-05-17

**Authors:** Tino Prell, Alexander Grimm, Hubertus Axer

**Affiliations:** ^1^Department of Geriatrics, Halle University Hospital, Halle, Germany; ^2^Department of Neurology, Jena University Hospital, Jena, Germany; ^3^Department of Neurology, Tübingen University Hospital, Tübingen, Germany

**Keywords:** sarcopenia, frailty, muscle thickness, cross-sectional area, ultrasound

## Abstract

Muscle ultrasound is a valuable non-invasive and cost-effective method in assessing muscle mass and structure, both of which are significant indicators for the development of sarcopenia and frailty in elderly individuals. Sarcopenia refers to the loss of muscle mass and strength that occurs with age, whereas frailty is a complex geriatric syndrome characterized by reduced physical function and an increased susceptibility to negative health outcomes. Both conditions are prevalent in older adults and are associated with higher risks of falls, disability, and mortality. By measuring muscle size and structure and several other ultrasound parameters, including muscle thickness, cross-sectional area, echogenicity (brightness in the ultrasound image), pennation angle, and fascicle length ultrasound can assist in identifying sarcopenia and frailty in older adults. In addition, ultrasound can be used to evaluate muscle function such as muscle contraction and stiffness, which may also be affected in sarcopenia and frailty. Therefore, muscle ultrasound could lead to better identification and tracking of sarcopenia and frailty. Such advancements could result in the implementation of earlier interventions to prevent or treat these conditions, resulting in an overall improvement in the health and quality of life of the elderly population. This narrative review describes the benefits and challenges when using ultra-sound for the evaluation of frailty and sarcopenia.

## Introduction

As people age, they are more prone to developing frailty (1, 2) and sarcopenia (3, 4). Frailty describes a condition of increased vulnerability to adverse health outcomes mainly due to age-associated decreased physical functions (5). Sarcopenia describes a reduction of strength and the loss of muscle mass with increasing age (6). Both can have a significant impact on an individual’s health and quality of life and commonly, they have been assessed through physical examination, anthropometric measures, and questionnaires (7). To effectively assess and treat frailty and sarcopenia, medical professionals must have an accurate and reliable way to measure them. Muscle ultrasound is a promising tool for this issue.

Muscle ultrasound is a patient-friendly, widely available, relatively inexpensive, fast and non-invasive imaging technique to create images of the body’s muscles (8). It can be used to measure muscle size and thickness as well as detect changes in muscle structure. Keeping track of body fat is an integral part of assessing nutrition and gauging any muscle loss. The repercussions of diminished muscle mass and functions have been brought into light in both short-term and long-term health issues. The validity and reliability of ultrasound has been subject of a recent systematic review (9), and ultrasound parameters have been shown to correlate well with other reference measures of muscle mass and various clinical outcomes (9, 10).

By using ultrasound to detect muscle loss and frailty in older adults, clinicians can identify patients at risk for complications or worsening health and provide appropriate prophylactic interventions before it is too late. Ultrasound can also be used to monitor changes in muscle status over time so that interventions can be monitored and adjusted accordingly. Further ultrasound is an easy available method to screen patients for underlying focal pathology concerning muscle damage, e.g., polyneuropathy, radiculopathy and focal myopathy.

This narrative review will discuss the use of muscle ultrasound in assessing sarcopenia and frailty, as well as highlights the benefits of using this technology for geriatric patients.

### Frailty

Frailty is an important geriatric syndrome (11–15). It is characterized by decreased physical function, reduced resilience, and increased vulnerability to adverse health out-comes (16). This complex geriatric syndrome is characterized by a decline in multiple physiological systems, including decreased muscle mass and strength, reduced energy levels, and decreased physical activity levels. This can lead to an increased risk of falls, fractures, and hospitalizations as well as to an increased risk of mortality. Frailty can be assessed by a variety of methods, including physical performance tests, laboratory tests, and questionnaires (7).

There are several definitions of frailty, including:

• The Fried phenotype (17): This definition of frailty is based on the criteria developed by Fried et al. in 2001. According to the cardiovascular health study (CHS) definition (18), the criteria include weakness, exhaustion, low physical activity, slow gait speed, and unintended weight loss.
• The Rockwood clinical frailty scale (RCFS) (19): This definition understands frailty as an accumulation of deficits. In a so-called frailty index, a large number of geriatric syndromes, functional limitations and social deficits are recorded and combined in a score. The Rockwood Frailty Index is therefore much more comprehensive than the Fried criteria.

As the elderly population grows, frailty has become a more common occurrence and can have a major influence on peoples’ quality of living. However, it can be challenging to accurately calculate its prevalence due to the variety of methods to measure frailty. To address this problem, Collard et al. (20) conducted a comprehensive review and meta-analysis of 21 studies to estimate the prevalence of frailty. The findings from the study revealed that the prevalence of frailty varied from 4.0 to 59.1%, with women having a higher rate than men. The prevalence reached its peak among those aged 85 years or older. These results are in line with those from the Survey of Health, Aging and Retirement in Europe (SHARE) project (21). As a result, early detection of frailty is critical for the timely start of preventive and curative procedures intended to decrease the load of geriatric syndromes and promoting healthy aging (1, 3, 16, 20, 22).

Regardless of the definition used, frailty is considered to be a complex geriatric syndrome that is associated with changes of muscular strength and function. This links frailty to another geriatric syndrome, i.e., sarcopenia.

### Sarcopenia

Sarcopenia is the age-related loss of muscle mass and strength with increasing prevalence in older adults (3, 23). This condition is a significant contributor to frailty and is associated with decreased physical function, increased risk of falls, and increased mortality. It is commonly associated with malnutrition. Numerous studies have indicated that sarcopenia is an independent predictor of poor clinical outcomes, including elevated risk of post-operative complications, falls, fractures, prolonged hospital stays, morbidity, and mortality (24–29). The prognosis of individuals with fractures or undergoing organ transplantation and major surgical interventions is worse if they are sarcopenic, which underlines the importance of assessing muscle and nutritional status to develop tailored and effective treatment strategies (28). A growing challenge in geriatric care is the increasing prevalence of sarcopenic obesity, because it is associated with greater disability, morbidity, and mortality than the two entities (obesity and sarcopenia) considered separately (29). It seems that muscle ultrasound is also a feasible and valuable tool to detect sarcopenic obesity, however, more research is needed for this topic (30, 31).

In general, the diagnosis of sarcopenia must include the evaluation of both muscle mass and muscle function (32). Accordingly, the European Working Group on Sarcopenia in Older People (EWGSOP2) criteria for sarcopenia are: low muscle mass, reduced muscle strength, and reduced physical performance (3, 10). To evaluate muscle mass, several methods, like dual x-ray absorptiometry (DXA), bioelectrical impedance analysis (BIA), computerized tomography (CT), and magnetic resonance imaging (MRI) are available. Additionally to the typical issues of reliability and validity, many of these methods used to measure muscle mass have significant shortcomings that make them inadequate for frequent use in elderly and frail individuals. Hence, the EWGSOP acknowledges the advantages of muscle sonography in diagnosing sarcopenia (3, 33), and suggests that it should be used in conjunction with other clinical tests such as assessing muscle strength and physical capability to provide a comprehensive understanding of muscle health in elderly.

### Muscle ultrasound

Muscle ultrasound is a non-invasive, widely available, inexpensive, and painless method to image the muscle. It is a valuable tool to assess muscle status in a variety of neuromuscular diseases (8, 34–36). Muscle ultrasound can assess different parameters of the muscle (37, 38): Muscle volume can be estimated by measuring longitudinal or oblique diameters or muscular cross-sectional area (CSA). Muscles can be normal, atrophic, or hypertrophic. As individual preconditions are quite heterogeneous, muscle volume estimates are mainly reliable in longitudinal examinations.

Muscle structure is mainly represented by the echogenicity of the muscle. The normal muscle structure shows low echogenicity. Fibroadipose septae and perimysium have a high echogenicity. The muscle is surrounded by echo-intense epimysium. In cross section, the normal muscle shows a starry night appearance with small echogenic dots and dashes. In longitudinal section, the muscle shows parallel echogenic lines along the longitudinal axis like a herringbone pattern. Muscle echogenicity depends on the contingent of connective tissue, the orientation of muscle fibers, the subcutaneous fat, age, and sex. Echogenicity can be measured semi-quantitatively using the Heckmatt score (39) or quantitatively using gray scale analysis (40).

Dynamic muscle ultrasound can image muscle contractions or spontaneous activity such as fasciculations. Doppler imaging helps to evaluate muscle vascularization. Individual parameters as sex, body mass index, height and weight might influence these measurements and must be borne in mind when interpreting muscle ultrasound.

With special focus on the elderly population muscle ultrasound has been mainly used to asses muscle mass. Ultrasound is a reliable and valid tool for the assessment of muscle size in older adults, including those with comorbid conditions (10). Ultrasound was shown to have good validity to estimate muscle mass as compared to DXA, MRI and CT (3, 41–48).

Recent studies have suggested that muscle ultrasound is most suitable to reliably assess muscle wasting in older people (49). Muscle ultrasound is particularly effective for measuring muscle size in different body parts, with the quadriceps femoris muscle (MQ) being one of the most reliable muscle groups to evaluate (50, 51). Moreover, it has been found to be useful in assessing muscle hypertrophy and atrophy by providing a direct insight into muscle size (52).

In the context of geriatric individuals, muscle ultrasound serves as a valuable means of evaluating muscle mass and structure. Additionally, it may offer insights into muscle function to some extent. Studies have revealed that the muscle power of the elderly is linked to the thickness of the vastus medialis (53), as well as to the echogenicity of the rectus femoris muscle measured by the mean value of pixel intensities (54, 55). Furthermore, parameters such as the pennation angle and muscle fascicle length, as evaluated while at rest or when contracted, are significantly associated with half of the decline in maximum force and shortening velocity of muscle fibers associated with sarcopenia (55).

## Assessment of sarcopenia by means of muscle ultrasound

Several studies have provided evidence that muscle ultrasound may be a valuable tool to reliably and validly assess sarcopenia (56–58). This technique can be utilized to quantify muscle size and structure, aiding in the detection of muscle wasting. Furthermore, it can be used to check muscle strength and contractility, which can be a sign of sarcopenia.

In order to perform comprehensive and comparative studies, it is essential to standardize the techniques and metrics utilized. This was achieved with the first SARCUS (SARCopenia through UltraSound) publication regarding the standardization of ultrasound muscle evaluation (58), which suggested consensus-based anatomical landmarks and features. Moreover, an instructional video (59) illustrated the five main parameters to be collected: muscle thickness, pennation angle, fascicle length, echo-intensity, and CSA. The second SARCUS article presented updated recommendations and provided additional anatomical landmarks and measuring points for more muscles (56). While numerous studies have been conducted on large muscle groups (e.g., quadriceps muscle), even smaller muscles may have outstanding importance as these have specific functions (e.g., the pharyngeal muscles or the diaphragm). The updated SARCUS review found that different approaches for ultrasound assessment exist for 39 muscles, which can influence the values measured (56). Sonographic parameters are shown in Table 1. It also amended the previous recommendations with four additional parameters for muscle assessment: muscle volume, muscle stiffness, contraction potential, and microcirculation (56).

**TABLE 1:** Sonographic parameters to assess muscle status in older patients.

Parameter	Definition (56)	Site of assessment (62)	References
Muscle thickness (MT)	Distance between deep and superficial aponeurosis	Every muscular compartment (most studies performed on quadriceps femoris)	(41, 42, 64)
Cross-sectional area (CSA)	Anatomical cross-sectional area (ACSA) was defined as the CSA of a muscle perpendicular to its longitudinal axis. Physiological cross-sectional area (PCSA) was defined as the CSA of a muscle perpendicular to its fibers.	ACSA: Lower limb muscular compartments (mostly rectus femoris muscle) PCSA: Quadriceps femoris	(65, 66)
Pennation angle (PA)	Angle of insertion of muscle fiber fascicles into the deep aponeurosis. It is closely related to the force-generating capacity of the muscle	Pennate muscles of the lower limb (mostly gastrocnemius medialis)	(56, 67)
Echo intensity (EI)	Brightness of the image acquired through ultrasound. It is expressed in gray scales (0–255).	Every muscular compartment (mostly quadriceps femoris)	(42, 68–75)
Fascicle length (Lf)	Length of the fascicular path between the insertions of the fascicle into the superficial and deep aponeuroses. Also formulas exist to calculate fascicle length (67, 76).	–	(68, 76–79)
Muscle volume (MV)	MV = 0.3∗MT+30.5∗LL, with MV = muscle volume, MT = muscle thickness and LL = limb length	–	(79)
Muscle contraction	Correlating the CSA in rest to the CSA in maximal contraction	–	(80, 81)

In particular, the value of muscle ultrasound to detect muscle stiffness is interesting, although the effect of aging on muscular stiffness is not fully clear (60). The interaction among the components of muscle mass influences the overall muscle stiffness, which is the range between maximal possible muscle distension and compaction (61). This is influenced by connective tissues like collagen in the extracellular matrix, giving rise to passive and active tension (62). Shear wave elastography (SWE) can evaluate the stiffness of the tissue quantitatively and non-invasively. Consequently, shear wave velocity was studied in the quadriceps muscles, hamstrings and biceps brachii muscles of volunteers of different age groups (26 young, 21 middle-aged and 30 elderly) (63). It was shown that total resting muscle shear wave velocity slowly decreased with age but a statistically significant reduction was only demonstrated in the elderly group. Muscle stiffness was 16.5% lower in the elderly group than in the young aged group, suggesting that SWE-measured muscle stiffness may be associated to muscle weakness.

However, several challenges remain when using ultrasound for detection of sarcopenia (82). It may not be possible to measure all ultrasonographic parameters in certain muscles or muscle groups, such as the diaphragm, where it is not possible to calculate the cross-sectional area. This is a limitation that cannot be addressed, and further research is needed to determine which components are the most significant. It is also critical to take gender-specific adjustments for regional muscle measurements into account when dealing with sarcopenia. The diagnostic work-up using ultrasound has to be limited to few skeletal muscles, and, thus, the most relevant muscles should be considered in sarcopenia (83).

Sarcopenia may impair different muscle groups heterogeneously since postural muscles are more affected than the nonpostural muscles (84, 85). Certain muscles, such as those in the anterior thigh and abdomen, tend to experience earlier atrophy than others (86). This means that in the early stages of sarcopenia, muscle loss in the lower body may be the first to occur (86). In particular, the anterior thigh muscles play an important role in daily life activities like standing, transferring, and climbing stairs. For this reason, it is necessary not only to evaluate general muscle loss, but also to evaluate the focused loss of muscle mass in localized areas most sensitive for sarcopenia in order to make an early and accurate diagnosis (87, 88). Thus, assessing the total muscle mass alone may not be efficient to diagnose sarcopenia, as normal or even higher muscle mass in the upper body can obscure the evaluation (89).

Finally, to date there are no established thresholds for ultrasound parameters to define low muscle mass or sarcopenia (90). A few studies proposed criteria to diagnose sarcopenia (91, 92); however, these need replication and validation in future studies. For instance, Minetto et al. (92) defined normative values of muscle thickness for the rectus femoris muscle (20 mm in men and 16 mm in women), the vastus lateralis (17 mm in men and 15 mm in women), and for the medial gastrocnemius muscle (13 mm in both men and women) (92).

Several investigations have revealed that specific ultrasound measurements of muscle tissue such muscle thickness and cross-sectional area can effectively identify sarcopenia in elderly people. Several smaller studies focused on distinct muscles and conditions, with the rectus femoris or anterior thigh muscles being the muscles studied most often (93–97). Matsuzawa et al. (97) conducted a study on hemodialysis patients and observed a considerable capacity of ultrasound to detect patients with an increased risk for sarcopenia (97). By using bioelectrical impedance analysis as reference method to evaluate muscle mass, they concluded that ultrasound-derived rectus femoris cross-sectional area was significantly correlated with muscle mass parameters and knee extensor muscle strength.

Tada et al. (98) proposed cut-off values for anterior thigh ultrasound examinations to distinguish between sarcopenia and obesity in patients with rheumatoid arthritis (98). Rustani et al. (99) provided cut-off values in geriatric outpatients and put forward rectus femoris muscle thickness as a suitable metric for sarcopenia screening. Yuguchi et al. (100) proposed a cut-off value of 11.6 mm for gastrocnemius muscle thickness (sensitivity, 0.83; specificity, 0.73) in elderly healthy Japanese based on the low skeletal muscle index derived from bioelectrical impedance analysis without distinguishing between sexes.

Hida et al. (101) conducted research on 201 individuals undergoing annual health screenings, relying mainly on BIA to measure muscle mass. The authors suggested distinct cut-off values for thigh muscle thickness (rectus femoris muscle and vastus intermedius muscle) for both sexes.

Sengul Aycicek et al. (102) provided normative values for the thickness the gastrocnemius muscle to likely identify sarcopenia in geriatric outpatients. The best cut-off value was defined to be ≤ 12.3 mm in women and ≤ 12.3 mm in men (102). In addition, it was demonstrated recently that the thickness of gastrocnemius, rectus femoris, and rectus abdominis muscles, as well as the CSA of rectus femoris are suitable to accurately predict sarcopenia (103). The suggested cut-off values were 13.9/13.8 mm (AUC: 0.817/0.707 mm) for the gastrocnemius muscle, 13/15.5 mm (AUC: 0.760/0.736 mm) for the rectus femoris muscle, and rectus abdominis muscle thickness {6.6/7.0 mm (AUC: 0.740/0.688 mm), and 4.3/5.2 cm^2^ (AUC: 0.766/0.773 cm^2^) for the rectus femoris CSA [for women and for men, respectively (103)]}.

A study conducted by Ata et al. (87) on 145 healthy individuals demonstrated that abdominal and thigh muscles were thinner in the age group ≥ 50 years, while the triceps muscle was thicker. Therefore, it was concluded that the anterior thigh and abdominal muscles were more susceptible to the effects of aging, while the upper extremity muscles were less affected (85).

Variations in muscle measurements for a particular individual may be determined by various factors such as age, gender, weight, and height. Consequently, it is necessary to take adjusted regional muscle measurements into account. For this purpose, the size of the thigh muscles has been related to body weight and height. Furthermore, sarcopenia could be identified by the ratio of thigh muscle cross-sectional area to body weight falling below two standard deviations of the young population (104).

Recently, the Society of Physical and Rehabilitation Medicine special interest group on sarcopenia (ISarcoPRM) has adopted the measurement of quadriceps muscle thickness as a diagnostic criterion for assessing low muscular mass (105). It was recommended to adjust muscle ultrasound thickness of the anterior thigh muscles for body mass index for the diagnosis of sarcopenia. Thus, the sonographic thigh adjustment ratio (STAR) was computed by dividing anterior thigh muscle thickness by body mass index. Kara et al. (88) defined sonographic thigh adjustment ratio cut-off values of 1.4 for males and 1.0 for females, which is two standard deviations below the mean values of healthy young adults. STAR values were negatively correlated with the Chair Stand Test and the Timed Up and Go Test in both sexes (88).

The ultrasound studies related to sarcopenia as discussed above are summarized in Supplementary Table 2.

## Muscle ultrasound used for assessment of frailty

To a less extent, muscle ultrasound has also been used to assess frailty. One study examined the association between a Point-of-Care ultrasonic (PoCUS) measure of muscle thickness and commonly used frailty measures in 177 older adults (age ≥ 65) (106). The results showed a weak inverse correlation between muscle thickness and the cardiovascular health study index, while no association was found between muscle thickness and the Rockwood clinical frailty scale. Therefore, screening for frailty by means of one single ultrasonic measure have to be done with caution (106).

Another study included 49 elderly people who were referred to an emergency medical service (107). Thigh muscle thickness was related to thigh length (U/S_whole/L_ index) and the Canadian Study of Healthy Aging Clinical Frailty Scale was used to evaluate frailty status. Here, increasing frailty score correlated with decreasing U/S_whole/L_ index for the whole muscle, and its two components–the rectus femoris and the vastus intermedius muscle as well. Although the explained variance of frailty ranged between 0.1 and 0.13 only, this suggests that frailty increases with the reduction of the thigh muscle mass of the patient (107).

Another smaller study compared preoperative ultrasound and CT-derived muscle measurements in people with frailty (18 patients) and without frailty (14 patients and 20 healthy volunteers) (108). Prior to surgery, ultrasound was used to measure the quadriceps and rectus femoris thickness, CT scans were used to measure psoas muscle cross-sectional area, and the Fried phenotype assessment was used to determine frailty. Results from receiver operating characteristic curve analysis revealed that sonographic quadriceps muscle thickness and CT-based psoas muscle CSA may be suitable to identify frailty (AUC, 0.80 [95% CI, 0.64 to 0.97] and 0.88 [95% CI, 0.76 to 1.00]), respectively.

Shah et al. (109) compared ultrasound muscle measurements with the FRAIL scale in 65 older adult trauma patients (65 years or older) in the emergency department with 15 patients who were considered frail based on the FRAIL scale. The ultrasound of the biceps and quadriceps muscles showed moderate agreement (diagnostic accuracy of 0.75) when compared to the reference standard (109).

Another study was conducted in 223 haemodialysis patients to study the association between ultrasound-derived bilateral anterior thigh thickness (BATT), sarcopenia, and frailty by utilizing common frailty tools such as the Frailty Phenotype, Frailty Index, Edmonton Frailty, and Clinical Frailty Scale (110). Ultrasound measurements of quadriceps muscle thickness were found to be variably associated with frailty depending on the frailty tool used (110).

Finally, a smaller study of 43 older adults (mean age of 78.5) could not find statistically significant differences of the ultrasound readings of upper arm muscles, quadriceps muscles, and abdominal wall muscles thickness between frail and non-frail subjects (111). However, in frail individuals a larger asymmetry of the biceps muscles was found, and a logistic regression model using the average quadriceps muscle thickness and biceps brachii muscle asymmetry could accurately identify frail patients (AUC of 0.816) indicating a mild to moderate association with frailty (111).

Supplementary Table 2 summarizes the ultrasound studies related to frailty, which were discussed here.

## Discussion and conclusion

Muscle ultrasound is an effective method for assessing sarcopenia. A recent meta-analysis (112) of diagnostic test accuracy of the use of muscle ultrasound to diagnose sarcopenia showed moderate diagnostic accuracy using muscle thickness of the gastrocnemius, rectus femoris, tibialis anterior, soleus, rectus abdominis and geniohyoid muscles, using the CSA of rectus femoris, biceps brachii muscles and gastrocnemius fascicle length. Many studies exist addressing the use of muscle ultrasound in sarcopenia (see Supplementary Table 1). However, reference standards, study populations, and ultrasound measurement methods were heterogeneous across the studies which impede the recommendation of a gold standard (113). Moreover, given the fact that sarcopenia has a timely and anatomic distribution (84–86) the sonographic evaluation of one target muscle should not be sufficient to proper assess the whole situation. The development of a compound muscle ultrasound score based on a standardized evaluation of different muscle groups as it is already established for peripheral nerve ultrasound (114) possibly would be of additional use.

Although the number of studies is still quite low, growing evidence suggests that ultrasound can also help to diagnose frailty (see Supplementary Table 2). However, as discussed above frailty is a complex geriatric syndrome, which is characterized by a decline in multiple physiological systems. Thus, decreased muscle mass and strength only partly contribute to frailty besides reduced energy levels, decreased physical activity levels, and others. Thus, evaluation of muscle status evaluated by means of ultrasound can necessarily represent only a part of the situation. Given the heterogeneous definitions and assessments of frailty further research is necessary to determine the use of ultrasound for its detection.

The advantage of muscle ultrasound is that it is a non-invasive, safe, and painless procedure that can be used to measure muscle size, shape, and composition easily and fast. These measurements can help to identify a decline in muscle mass, strength, and size, which can help medical professionals identify and treat geriatric syndromes more effectively. Although reliability of intra-rater and inter-rater reliability of muscle ultrasound is rather good (10), individual variability in sonographic measurements may be due to individual constitutional differences, age, gender, exercise and training status, nutrition, and many others. Thus, interpretation of results may be hampered by multifactorial influencies, which may make it difficult to define rigor cut-off values.

As the population continues to age, it is important to have accurate and reliable methods for assessing geriatric syndromes. Muscle ultrasound has the potential to identify frailty and sarcopenia early and treat both more effectively. This is particularly relevant for people who are unable to provide accurate medical history (emergency rooms, people with dementia). Ultrasound can help to quickly stratify geriatric patients prognostically in these cases in a point of care setting.

No consensus has been reached yet with regard to which muscle group should be measured, the specific probe site, or the criteria for determining low muscle mass in frailty. The wide range of approaches for measuring and analyzing muscle mass could have an effect on future clinical and research use, so it is essential that a standardized and straightforward technique have to be defined, which is the major challenge in this field.

## Author contributions

TP: Conceptualization, Writing – original draft, Writing – review & editing. AG: Writing – review & editing. HA: Writing – review & editing.

## References

[1] Proietti M Cesari M. Frailty: What is it? *Adv Exp Med Biol.* (2020) 1216:1–7. 10.1007/9783-030-33330-0_1 31894541

[2] Hoogendijk EO Afilalo J Ensrud KE Kowal P Onder G Fried LP. Frailty: Implications for clinical practice and public health. *Lancet.* (2019) 394:1365–75. 10.1016/S0140-6736(19)31786-6 31609228

[3] Cruz-Jentoft AJ Bahat G Bauer J Boirie Y Bruyère O Cederholm T Sarcopenia: Revised European consensus on definition and diagnosis. *Age Ageing.* (2019) 48:16–31. 10.1093/ageing/afy169 30312372 PMC6322506

[4] Naruse M Trappe S Trappe TA. Human skeletal muscle-specific atrophy with aging: A comprehensive review. *J Appl Physiol.* (2023) 134:900– 914. 10.1152/japplphysiol.00768.2022 36825643 PMC10069966

[5] Xue Q-L. The frailty syndrome: Definition and natural history. *Clin Geriatr Med.* (2011) 27:1–15. 10.1016/j.cger.2010.08.009 21093718 PMC3028599

[6] Sieber CC. Malnutrition and sarcopenia. *Aging Clin Exp Res.* (2019) 31:793–8. 10.1007/s40520-019-01170-1 31148100

[7] Wleklik M Uchmanowicz I Jankowska EA Vitale C Lisiak M Drozd M Multidimensional approach to frailty. *Front Psychol.* (2020) 11:564. 10.3389/fpsyg.2020.00564 32273868 PMC7115252

[8] Wijntjes J van Alfen N. Muscle ultrasound: Present state and future opportunities. *Muscle Nerve.* (2021) 63:455–66. 10.1002/mus.27081 33051891 PMC8048972

[9] Casey P Alasmar M McLaughlin J Ang Y McPhee J Heire P The current use of ultrasound to measure skeletal muscle and its ability to predict clinical outcomes: A systematic review. *J Cachexia Sarcopenia Muscle.* (2022) 13:2298–309. 10.1002/jcsm.13041 35851996 PMC9530572

[10] Nijholt W Scafoglieri A Jager-Wittenaar H Hobbelen JSM van der Schans CP. The reliability and validity of ultrasound to quantify muscles in older adults: A systematic review. *J Cachexia Sarcopenia Muscle.* (2017) 8:702–12. 10.1002/jcsm.12210 28703496 PMC5659048

[11] Inouye SK Studenski S Tinetti ME Kuchel GA. Geriatric syndromes: Clinical, research, and policy implications of a core geriatric concept. *J Am Geriatr Soc.* (2007) 55:780–91. 10.1111/j.1532-5415.2007.01156.x 17493201 PMC2409147

[12] Carlson C Merel SE Yukawa M. Geriatric syndromes and geriatric assessment for the generalist. *Med Clin North Am.* (2015) 99:263–79. 10.1016/j.mcna.2014.11.003 25700583

[13] Veronese N Custodero C Demurtas J Smith L Barbagallo M Maggi S Comprehensive geriatric assessment in older people: An umbrella review of health outcomes. *Age Ageing.* (2022) 51:afac104. 10.1093/ageing/afac104 35524746

[14] Magnuson A Sattar S Nightingale G Saracino R Skonecki E Trevino KMA. Practical guide to geriatric syndromes in older adults with cancer: A focus on falls, cognition, polypharmacy, and depression. *Am Soc Clin Oncol Educ Book.* (2019) 39:e96–109. 10.1200/EDBK_237641 31099668 PMC12452041

[15] Walston J Buta B Xue Q-L. Frailty screening and interventions: Considerations for clinical practice. *Clin Geriatr Med.* (2018) 34:25–38. 10.1016/j.cger.2017.09.004 29129215 PMC5726589

[16] Cesari M Prince M Thiyagarajan JA De Carvalho IA Bernabei R Chan P Frailty: An emerging public health priority. *J Am Med Dir Assoc.* (2016) 17:188–92. 10.1016/j.jamda.2015.12.016 26805753

[17] Fried LP Tangen CM Walston J Newman AB Hirsch C Gottdiener J Frailty in older adults: Evidence for a phenotype. *J Gerontol A Biol Sci Med Sci.* (2001) 56:M146–56. 10.1093/gerona/56.3.m146 11253156

[18] Fried LP Borhani NO Enright P Furberg CD Gardin JM Kronmal RA The cardiovascular health study: Design and rationale. *Ann Epidemiol.* (1991) 1:263–76. 10.1016/1047-2797(91)90005-w 1669507

[19] Rockwood K Song X MacKnight C Bergman H Hogan DB McDowell I A global clinical measure of fitness and frailty in elderly people. *CMAJ.* (2005) 173:489–95. 10.1503/cmaj.050051 16129869 PMC1188185

[20] Collard RM Boter H Schoevers RA Oude Voshaar RC. Prevalence of frailty in community-dwelling older persons: A systematic review. *J Am Geriatr Soc.* (2012) 60:1487–92. 10.1111/j.1532-5415.2012.04054.x 22881367

[21] SHARE. 2023 Available online at: https://share-eric.eu/ (accessed February 25, 2023).

[22] Searle SD Rockwood K. What proportion of older adults in hospital are frail? *Lancet.* (2018) 391:1751–2. 10.1016/S0140-6736(18)30907-3 29706363

[23] Cruz-Jentoft AJ Landi F Schneider SM Zúñiga C Arai H Boirie Y Prevalence of and interventions for sarcopenia in ageing adults: A systematic review. Report of the international sarcopenia initiative (EWGSOP and IWGS). *Age Ageing.* (2014) 43:748–59. 10.1093/ageing/afu115 25241753 PMC4204661

[24] Yang Z Zhou X Ma B Xing Y Jiang X Wang Z. Predictive value of preoperative sarcopenia in patients with gastric cancer: A meta-analysis and systematic review. *J Gastrointest Surg.* (2018) 22:1890–902. 10.1007/s11605-018-3856-0 29987739

[25] Deng H-Y Zha P Peng L Hou L Huang K-L Li X-Y. Preoperative sarcopenia is a predictor of poor prognosis of esophageal cancer after esophagectomy: A comprehensive systematic review and meta-analysis. *Dis Esophagus.* (2019) 32:doy115. 10.1093/dote/doy115 30496385

[26] Pamoukdjian F Bouillet T Lévy V Soussan M Zelek L Paillaud E. Prevalence and predictive value of pre-therapeutic sarcopenia in cancer patients: A systematic review. *Clin Nutr.* (2018) 37:1101–13. 10.1016/j.clnu.2017.07.010 28734552

[27] Bokshan SL DePasse JM Daniels AH. Sarcopenia in orthopedic surgery. *Orthopedics.* (2016) 39:e295–300. 10.3928/01477447-20160222-02 26913764

[28] Lindström I Khan N Vänttinen T Peltokangas M Sillanpää N Oksala N. Psoas muscle area and quality are independent predictors of survival in patients treated for abdominal aortic aneurysms. *Ann Vasc Surg.* (2019) 56:183–193.e3. 10.1016/j.avsg.2018.08.096 30476615

[29] Donini LM Busetto L Bischoff SC Cederholm T Ballesteros-Pomar MD Batsis JA Definition and diagnostic criteria for sarcopenic obesity: ESPEN and EASO consensus statement. *Obes Facts.* (2022) 15:321–35. 10.1159/000521241 35196654 PMC9210010

[30] Deniz O Cruz-Jentoft A Sengul Aycicek G Unsal P Esme M Ucar Y Role of ultrasonography in estimating muscle mass in sarcopenic obesity. *JPEN J Parenter Enteral Nutr.* (2020) 44:1398–406. 10.1002/jpen.1830 32342544

[31] Simó-Servat A Ibarra M Libran M Rodríguez S Perea V Quirós C Usefulness of muscle ultrasound to study sarcopenic obesity: A pilot case-control study. *J Clin Med.* (2022) 11:2886. 10.3390/jcm11102886 35629009 PMC9143348

[32] Tosato M Marzetti E Cesari M Savera G Miller RR Bernabei R Measurement of muscle mass in sarcopenia: From imaging to biochemical markers. *Aging Clin Exp Res.* (2017) 29:19–27. 10.1007/s40520-016-0717-0 28176249

[33] Stringer HJ Wilson D. The role of ultrasound as a diagnostic tool for sarcopenia. *J Frailty Aging.* (2018) 7:258–61. 10.14283/jfa.2018.24 30298175 PMC6208738

[34] Pillen S van Alfen N. Skeletal muscle ultrasound. *Neurol Res.* (2011) 33:1016–24. 10.1179/1743132811Y.0000000010 22196753

[35] Pillen S Arts IMP Zwarts MJ. Muscle ultrasound in neuromuscular disorders. *Muscle Nerve.* (2008) 37:679–93. 10.1002/mus.21015 18506712

[36] Cartwright MS Demar S Griffin LP Balakrishnan N Harris JM Walker FO. Validity and reliability of nerve and muscle ultrasound. *Muscle Nerve.* (2013) 47:515–21. 10.1002/mus.23621 23400913

[37] Grimm A Axer H Quasthoff S. 27 Methodik muskelsonografie. In: Bischoff C Buchner H editors. *SOPs neurophysiologische diagnostik.* Stuttgart: Georg Thieme Verlag (2018). p. 185–8.

[38] Walker FO Cartwright MS. *Neuromuscular ultrasound.* Philadelphia, PA: WB Saunders (2011).

[39] Heckmatt JZ Leeman S Dubowitz V. Ultrasound imaging in the diagnosis of muscle disease. *J Pediatr.* (1982) 101:656–60. 7131136 10.1016/s0022-3476(82)80286-2

[40] Pillen S van Keimpema M Nievelstein RAJ Verrips A van Kruijsbergen-Raijmann W Zwarts MJ. Skeletal muscle ultrasonography: Visual versus quantitative evaluation. *Ultrasound Med Biol.* (2006) 32:1315–21. 10.1016/j.ultrasmedbio.2006.05.028 16965971

[41] Paris MT Lafleur B Dubin JA Mourtzakis M. Development of a bedside viable ultrasound protocol to quantify appendicular lean tissue mass. *J Cachexia Sarcopenia Muscle.* (2017) 8:713–26. 10.1002/jcsm.12213 28722298 PMC5659058

[42] Yamada M Kimura Y Ishiyama D Nishio N Abe Y Kakehi T Differential characteristics of skeletal muscle in community-dwelling older adults. *J Am Med Dir Assoc.* (2017) 18:807.e9–807.e16. 10.1016/j.jamda.2017.05.011 28676289

[43] Ismail C Zabal J Hernandez HJ Woletz P Manning H Teixeira C Diagnostic ultrasound estimates of muscle mass and muscle quality discriminate between women with and without sarcopenia. *Front Physiol.* (2015) 6:302. 10.3389/fphys.2015.00302 26578974 PMC4625057

[44] Berger J Bunout D Barrera G de la Maza MP Henriquez S Leiva L Rectus femoris (RF) ultrasound for the assessment of muscle mass in older people. *Arch Gerontol Geriatr.* (2015) 61:33–8. 10.1016/j.archger.2015.03.006 25890633

[45] Abe T Patterson KM Stover CD Geddam DAR Tribby AC Lajza DG Site-specific thigh muscle loss as an independent phenomenon for age-related muscle loss in middle-aged and older men and women. *Age (Dordr).* (2014) 36:9634. 10.1007/s11357-014-9634-3 24569919 PMC4082600

[46] Thomaes T Thomis M Onkelinx S Coudyzer W Cornelissen V Vanhees L. Reliability and validity of the ultrasound technique to measure the rectus femoris muscle diameter in older CAD-patients. *BMC Med Imaging.* (2012) 12:7. 10.1186/1471-2342-12-7 22471726 PMC3342139

[47] Seymour JM Ward K Sidhu PS Puthucheary Z Steier J Jolley CJ Ultrasound measurement of rectus femoris cross-sectional area and the relationship with quadriceps strength in COPD. *Thorax.* (2009) 64:418–23. 10.1136/thx.2008.103986 19158125

[48] Sipilä S Suominen H. Muscle ultrasonography and computed tomography in elderly trained and untrained women. *Muscle Nerve.* (1993) 16:294–300. 10.1002/mus.880160309 8446128

[49] Bencivenga L Picaro F Ferrante L Komici K Ruggiero F Sepe I Muscle ultrasound as imaging domain of frailty. *Front Med (Lausanne).* (2022) 9:922345. 10.3389/fmed.2022.922345 35899217 PMC9309884

[50] Guerri S Mercatelli D Aparisi Gómez MP Napoli A Battista G Guglielmi G Quantitative imaging techniques for the assessment of osteoporosis and sarcopenia. *Quant Imaging Med Surg.* (2018) 8:60–85. 10.21037/qims.2018.01.05 29541624 PMC5835658

[51] Naimo MA Varanoske AN Hughes JM Pasiakos SM. Skeletal muscle quality: A biomarker for assessing physical performance capabilities in young populations. *Front Physiol.* (2021) 12:706699. 10.3389/fphys.2021.706699 34421645 PMC8376973

[52] Minetto MA Busso C Gamerro G Lalli P Massazza G Invernizzi M. Quantitative assessment of volumetric muscle loss: Dual-energy X-ray absorptiometry and ultrasonography. *Curr Opin Pharmacol.* (2021) 57:148–56. 10.1016/j.coph.2021.02.002 33735662

[53] Madden KM Feldman B Arishenkoff S Meneilly GS. A rapid point-of-care ultrasound marker for muscle mass and muscle strength in older adults. *Age Ageing.* (2021) 50:505–10. 10.1093/ageing/afaa163 32909032 PMC7936023

[54] Strasser EM Draskovits T Praschak M Quittan M Graf A. Association between ultrasound measurements of muscle thickness, pennation angle, echogenicity and skeletal muscle strength in the elderly. *Age (Dordr).* (2013) 35:2377–88. 10.1007/s11357-013-9517-z 23456136 PMC3824993

[55] Albano D Messina C Vitale J Sconfienza LM. Imaging of sarcopenia: Old evidence and new insights. *Eur Radiol.* (2020) 30:2199–208. 10.1007/s00330-019-06573-2 31834509

[56] Perkisas S Bastijns S Baudry S Bauer J Beaudart C Beckwée D Application of ultrasound for muscle assessment in sarcopenia: 2020 SARCUS update. *Eur Geriatr Med.* (2021) 12:45–59. 10.1007/s41999-020-00433-9 33387359

[57] Ticinesi A Meschi T Narici MV Lauretani F Maggio M. Muscle ultrasound and sarcopenia in older individuals: A clinical perspective. *J Am Med Dir Assoc.* (2017) 18:290–300. 10.1016/j.jamda.2016.11.013 28202349

[58] Perkisas S Baudry S Bauer J Beckwée D De Cock A-M Hobbelen H Application of ultrasound for muscle assessment in sarcopenia: Towards standardized measurements. *Eur Geriatr Med.* (2018) 9:739–57. 10.1007/s41999-018-0104-9 34674473

[59] SARCUS. *Instructional video 2019.* (2023). Available online at: https://www.youtube.com/watch?v=y4gSmxwvujE (accessed March 01, 2023).

[60] Brown M Fisher JS Salsich G. Stiffness and muscle function with age and reduced muscle use. *J Orthop Res.* (1999) 17:409–14. 10.1002/jor.1100170317 10376731

[61] Ikezoe T Asakawa Y Fukumoto Y Tsukagoshi R Ichihashi N. Associations of muscle stiffness and thickness with muscle strength and muscle power in elderly women. *Geriatr Gerontol Int.* (2012) 12:86–92. 10.1111/j.1447-0594.2011.00735.x 21883786

[62] Hug F Tucker K Gennisson J-L Tanter M Nordez A. Elastography for muscle biomechanics: Toward the estimation of individual muscle force. *Exerc Sport Sci Rev.* (2015) 43:125–33. 10.1249/JES.0000000000000049 25906424

[63] Alfuraih AM Tan AL O’Connor P Emery P Wakefield RJ. The Effect of Ageing on Shear Wave Elastography Muscle Stiffness in Adults. *Aging Clin Exp Res.* (2019) 31:1755–63. 10.1007/s40520-019-01139-0 30762201 PMC6825644

[64] Hagoort I Hortobágyi T Vuillerme N Lamoth CJC Murgia A. Age– and muscle-specific reliability of muscle architecture measurements assessed by two-dimensional panoramic ultrasound. *Biomed Eng Online.* (2022) 21:15. 10.1186/s12938-021-00967-4 35152889 PMC8842860

[65] Reeves ND Maganaris CN Narici MV. Ultrasonographic assessment of human skeletal muscle size. *Eur J Appl Physiol.* (2004) 91:116–8. 10.1007/s00421-003-0961-9 14639480

[66] Scanlon TC Fragala MS Stout JR Emerson NS Beyer KS Oliveira LP Muscle architecture and strength: Adaptations to short-term resistance training in older adults. *Muscle Nerve.* (2014) 49:584–92. 10.1002/mus.23969 23893353

[67] Morse CI Thom JM Reeves ND Birch KM Narici MV. In vivo physiological cross-sectional area and specific force are reduced in the gastrocnemius of elderly men. *J Appl Physiol.* (2005) 99:1050–5. 10.1152/japplphysiol.01186.2004 15905324

[68] Rech A Radaelli R Goltz FR da Rosa LHT Schneider CD Pinto RS. Echo intensity is negatively associated with functional capacity in older women. *Age (Dordr).* (2014) 36:9708. 10.1007/s11357-014-9708-2 25167965 PMC4453939

[69] Pillen S Tak RO Zwarts MJ Lammens MMY Verrijp KN Arts IMP Skeletal muscle ultrasound: Correlation between fibrous tissue and echo intensity. *Ultrasound Med Biol.* (2009) 35:443–6. 10.1016/j.ultrasmedbio.2008.09.016 19081667

[70] Cadore EL Izquierdo M Conceição M Radaelli R Pinto RS Baroni BM Echo intensity is associated with skeletal muscle power and cardiovascular performance in elderly men. *Exp Gerontol.* (2012) 47:473–8. 10.1016/j.exger.2012.04.002 22525196

[71] Fukumoto Y Ikezoe T Yamada Y Tsukagoshi R Nakamura M Mori N Skeletal muscle quality assessed from echo intensity is associated with muscle strength of middle-aged and elderly persons. *Eur J Appl Physiol.* (2012) 112:1519–25. 10.1007/s00421-011-2099-5 21847576

[72] Watanabe Y Yamada Y Fukumoto Y Ishihara T Yokoyama K Yoshida T Echo intensity obtained from ultrasonography images reflecting muscle strength in elderly men. *Clin Interv Aging.* (2013) 8:993–8. 10.2147/CIA.S47263 23926426 PMC3732157

[73] Akazawa N Okawa N Tamura K Moriyama H. Relationships between intramuscular fat, muscle strength and gait independence in older women: A cross-sectional study. *Geriatr Gerontol Int.* (2017) 17:1683–8. 10.1111/ggi.12869 27506895

[74] Kawai H Kera T Hirayama R Hirano H Fujiwara Y Ihara K Morphological and qualitative characteristics of the quadriceps muscle of community-dwelling older adults based on ultrasound imaging: Classification using latent class analysis. *Aging Clin Exp Res.* (2018) 30:283–91. 10.1007/s40520-017-0781-0 28577161

[75] Akima H Yoshiko A Tomita A Ando R Saito A Ogawa M Relationship between quadriceps echo intensity and functional and morphological characteristics in older men and women. *Arch Gerontol Geriatr.* (2017) 70:105–11. 10.1016/j.archger.2017.01.014 28126635

[76] Narici MV Maganaris CN Reeves ND Capodaglio P. Effect of aging on human muscle architecture. *J Appl Physiol.* (2003) 95:2229–34. 10.1152/japplphysiol.00433.2003 12844499

[77] Alonso-Fernandez D Gutierrez-Sanchez Á Garcia-Remeseiro T Garganta R. Effects of the nordic hamstring exercise on the architecture of the semitendinosus. *Isokinet Exerc Sci.* (2018) 26:81–8. 10.3233/IES-172196

[78] Kruse NT Hughes WE Casey DP. Mechanistic insights into the modulatory role of the mechanoreflex on central hemodynamics using passive leg movement in humans. *J Appl Physiol.* (2018) 125:545–52. 10.1152/japplphysiol.01085.2017 29771607 PMC6139517

[79] Merrigan JJ White JB Hu YE Stone JD Oliver JM Jones MT. Differences in elbow extensor muscle characteristics between resistance-trained men and women. *Eur J Appl Physiol.* (2018) 118:2359–66. 10.1007/s00421-018-3962-4 30097710

[80] Liu X Zhang X Feng Q. Dynamic change in the thickness of the masseter muscle between contraction and relaxation is associated with the masticatory function in older adults: A cross-sectional study. *Ann Palliat Med.* (2022) 11:3755–63. 10.21037/apm-22-1344 36636000

[81] Kelp NY Gore A Clemente CJ Tucker K Hug F Dick TJM. Muscle architecture and shape changes in the gastrocnemii of active younger and older adults. *J Biomech.* (2021) 129:110823. 10.1016/j.jbiomech.2021.110823 34736086

[82] Ticinesi A Meschi T Maggio M Narici MV. Application of ultrasound for muscle assessment in sarcopenia: The challenge of implementing protocols for clinical practice. *Eur Geriatr Med.* (2019) 10:155–6. 10.1007/s41999-018-0126-3 32720272

[83] Ata AM Kara M Özçakar L. Ultrasound imaging/measurements for skeletal muscles in sarcopenia: An aide memoire. *Eur Geriatr Med.* (2021) 12:425–6. 10.1007/s41999-021-00461-z 33575882

[84] Narici MV Maffulli N. Sarcopenia: Characteristics, mechanisms and functional significance. *Br Med Bull.* (2010) 95:139–59. 10.1093/bmb/ldq008 20200012

[85] Mitchell WK Williams J Atherton P Larvin M Lund J Narici M. Sarcopenia, dynapenia, and the impact of advancing age on human skeletal muscle size and strength; A quantitative review. *Front Physiol.* (2012) 3:260. 10.3389/fphys.2012.00260 22934016 PMC3429036

[86] Abe T Loenneke JP Thiebaud RS Ogawa M Mitsukawa N. Age-related site-specific muscle loss in the thigh and zigzag walking performance in older men and women. *Acta Physiol Hung.* (2014) 101:488–95. 10.1556/APhysiol.101.2014.006 25201711

[87] Ata AM Kara M Kaymak B Gürçay E Çakır B Ünlü H Regional and total muscle mass, muscle strength and physical performance: The potential use of ultrasound imaging for sarcopenia. *Arch Gerontol Geriatr.* (2019) 83:55–60. 10.1016/j.archger.2019.03.014 30953961

[88] Kara M Kaymak B Ata AM Özkal Ö Kara Ö Baki A STAR–sonographic thigh adjustment ratio: A golden formula for the diagnosis of sarcopenia. *Am J Phys Med Rehabil.* (2020) 99:902. 10.1097/PHM.0000000000001439 32941253

[89] Abe T Thiebaud RS Loenneke JP Loftin M Fukunaga T. Prevalence of site-specific thigh sarcopenia in Japanese men and women. *Age (Dordr).* (2014) 36:417–26. 10.1007/s11357-013-9539-6 23686131 PMC3889892

[90] Wang J-C Wu W-T Chang K-V Chen L-R Chi S-Y Kara M Ultrasound imaging for the diagnosis and evaluation of sarcopenia: An umbrella review. *Life.* (2022) 12:9. 10.3390/life12010009 35054402 PMC8781401

[91] Abe T Counts BR Barnett BE Dankel SJ Lee K Loenneke JP. Associations between handgrip strength and ultra-sound-measured muscle thickness of the hand and forearm in young men and women. *Ultrasound Med Biol.* (2015) 41:2125–30. 10.1016/j.ultrasmedbio.2015.04.004 25959055

[92] Minetto MA Caresio C Menapace T Hajdarevic A Marchini A Molinari F Ultrasound-based detection of low muscle mass for diagnosis of sarcopenia in older adults. *PM R.* (2016) 8:453–62. 10.1016/j.pmrj.2015.09.014 26431809

[93] Lee Z-Y Ong SP Ng CC Yap CSL Engkasan JP Barakatun-Nisak MY Association between ultrasound quadriceps muscle status with premorbid functional status and 60-day mortality in mechanically ventilated critically ill patient: A single-center prospective observational study. *Clin Nutr.* (2021) 40:1338–47. 10.1016/j.clnu.2020.08.022 32919818

[94] Bury C DeChicco R Nowak D Lopez R He L Jacob S Use of bedside ultrasound to assess muscle changes in the critically ill surgical patient. *JPEN J Parenter Enteral Nutr.* (2021) 45:394–402. 10.1002/jpen.1840 32391964

[95] Rodrigues CN Ribeiro Henrique J Ferreira ÁRS Correia MITD. Ultrasonography and other nutrition assessment methods to monitor the nutrition status of critically ill patients. *JPEN J Parenter Enteral Nutr.* (2021) 45:982–90. 10.1002/jpen.1966 32700339

[96] Er B Simsek M Yildirim M Halacli B Ocal S Ersoy EO Association of baseline diaphragm, rectus femoris and vastus intermedius muscle thickness with weaning from mechanical ventilation. *Respir Med.* (2021) 185:106503. 10.1016/j.rmed.2021.106503 34166958

[97] Matsuzawa R Yamamoto S Suzuki Y Imamura K Harada M Matsunaga A The clinical applicability of ultrasound technique for diagnosis of sarcopenia in hemodialysis patients. *Clin Nutr.* (2021) 40:1161–7. 10.1016/j.clnu.2020.07.025 32798065

[98] Tada M Yamada Y Mandai K Hidaka N. Screening for sarcopenia and obesity by measuring thigh muscle and fat thickness by ultrasound in patients with rheumatoid arthritis. *Osteoporos Sarcopenia.* (2021) 7:81–7. 10.1016/j.afos.2021.06.002 34278004 PMC8261725

[99] Rustani K Kundisova L Capecchi PL Nante N Bicchi M. Ultrasound measurement of rectus femoris muscle thickness as a quick screening test for sarcopenia assessment. *Arch Gerontol Geriatr.* (2019) 83:151–4. 10.1016/j.archger.2019.03.021 31029040

[100] Yuguchi S Asahi R Kamo T Azami M Ogihara H. Gastrocnemius thickness by ultrasonography indicates the low skeletal muscle mass in Japanese elderly people. *Arch Gerontol Geriatr.* (2020) 90:104093. 10.1016/j.archger.2020.104093 32526562

[101] Hida T Ando K Kobayashi K Ito K Tsushima M Kobayakawa T Ultrasound measurement of thigh muscle thickness for assessment of sarcopenia. *Nagoya J Med Sci.* (2018) 80:519–27. 10.18999/nagjms.80.4.519 30587866 PMC6295433

[102] Sengul Aycicek G Ozsurekci C Caliskan H Kizilarslanoglu MC Tuna Dogrul R Balci C Ultrasonography versus bioelectrical impedance analysis: Which predicts muscle strength Bet-ter? *Acta Clin Belg.* (2021) 76:204–8. 10.1080/17843286.2019.1704989 31847723

[103] Ozturk Y Koca M Burkuk S Unsal P Dikmeer A Oytun MG The role of muscle ultrasound to predict sarcopenia. *Nutrition.* (2022) 101:111692. 10.1016/j.nut.2022.111692 35660496

[104] Kim TN Park MS Lee EJ Chung HS Yoo HJ Kang HJ Comparisons of three different methods for defining sarcopenia: An aspect of cardiometabolic risk. *Sci Rep.* (2017) 7:6491. 10.1038/s41598-017-06831-7 28747657 PMC5529503

[105] Kara M Kaymak B Frontera WR Ata AM Ricci V Ekiz T Diagnosing sarcopenia: Functional perspectives and a new algorithm from ISarcoPRM. *J Rehabil Med.* (2021) 53:2806. 10.2340/16501977-2851 34121127 PMC8814891

[106] Madden KM Feldman B Arishenkoff S Meneilly GS. Point-of-care ultrasound measures of muscle and frailty measures. *Eur Geriatr Med.* (2021) 12:161–6. 10.1007/s41999-020-00401-3 32960448

[107] Salim SY Al-Khathiri O Tandon P Baracos VE Churchill TA Warkentin LM Thigh ultrasound used to identify frail elderly patients with sarcopenia undergoing surgery: A pilot study. *J Surg Res.* (2020) 256:422–32. 10.1016/j.jss.2020.06.043 32795705

[108] Canales C Mazor E Coy H Grogan TR Duval V Raman S Preoperative point-of-care ultrasound to identify frailty and predict postoperative outcomes: A diagnostic accuracy study. *Anesthesiology.* (2022) 136:268–78. 10.1097/ALN.0000000000004064 34851395 PMC9843825

[109] Shah SP Penn K Kaplan SJ Vrablik M Jablonowski K Pham TN Comparison of bedside screening methods for frailty assessment in older adult trauma patients in the emergency department. *Am J Emerg Med.* (2019) 37:12–8. 10.1016/j.ajem.2018.04.028 29728285

[110] Anderson BM Wilson DV Qasim M Correa G Evison F Gallier S Ultrasound quadriceps muscle thickness is variably associated with frailty in haemodialysis recipients. *BMC Nephrol.* (2023) 24:16. 10.1186/s12882-022-03043-8 36653750 PMC9847024

[111] Benton E Liteplo AS Shokoohi H Loesche MA Yacoub S Thatphet P A pilot study examining the use of ultrasound to measure sarcopenia, frailty and fall in older patients. *Am J Emerg Med.* (2021) 46:310–6. 10.1016/j.ajem.2020.07.081 33041131

[112] Fu H Wang L Zhang W Lu J Yang M. Diagnostic test accuracy of ultrasound for sarcopenia diagnosis: A systematic review and meta-analysis. *J Cachexia Sarcopenia Muscle.* (2023) 14:57–70. 10.1002/jcsm.13149 36513380 PMC9891970

[113] Mombiela RM Vucetic J Rossi F Tagliafico AS. Ultrasound biomarkers for sarcopenia: What can we tell so far? *Semin Musculoskelet Radiol.* (2020) 24:181–93. 10.1055/s-0039-3402745 32438444

[114] Grimm A Rattay TW Winter N Axer H. Peripheral nerve ultrasound scoring systems: Benchmarking and comparative analysis. *J Neurol.* (2017) 264:243–53. 10.1007/s00415-016-8305-y 27878436

