# Supplementary material for: Uncovering sarcopenia and frailty in older adults by using muscle ultrasound—A narrative review
Source: Front Med (Lausanne). 2024 May 17;11:1333205. doi: 10.3389/fmed.2024.1333205 (PMC11140070; doi:10.3389/fmed.2024.1333205)
Supplement: Supplementary file 1 [file Table_1.DOCX]

**Supplementary Table S1.** Muscle ultrasound studies associated to sarcopenia.

| Reference | Study population | Parameters measured | Results related to muscle ultrasound | Conclusions |
| --- | --- | --- | --- | --- |
| Narici et al., 1985 (77) | 14 young (27-42 years) and on 16 older (aged 70-81 years) physically active men | - Computerized tomography (CT): gastrocnemius muscle cross-sectional area and volume - Muscle ultrasound: gastrocnemius fascicle length (Lf) and pennation angle (theta) | All muscle architectural parameters were reduced in the elderly compared with the younger adults  Lf and theta were smaller in the elderly group by 10.2% (p < 0.01) and 13.2% (p < 0.01), respectively | aging significantly affects human skeletal muscle architecture |
| Sipilä and Suominen, 1995 (48) | 21 female athletes and 15 controls, aged 66-85 years | - Muscle ultrasound: cross-sectional area (CSA) - CT: CSA | 30% higher values in CT measurements  athletes had larger quadriceps CSA and more discerned fasciae and connective tissue septa but less fat than the controls | Muscle ultrasound and CT are useful methods in comparing muscle mass and structure in elderly trained and untrained women |
| Seymour et al., 2009 (47) | 26 healthy volunteers of 63±9 years and 30 patients with COPD of 67±9 years age | - Muscle ultrasound: Rectus femoris muscle cross-sectional area (CSA) - Electrical bioimpedance: whole-body fat-free mass (FFM) - Quadriceps strength by maximum voluntary contraction - Twitch tension following magnetic femoral nerve stimulation. | CSA of rectus femoris muscle was reduced in patients with COPD by 25% of the mean value in healthy subjects (-115 mm^2^) and was related to MRC dyspnoea scale score | Ultrasound of rectus femoris is an effort-independent and radiation-free method of measuring quadriceps muscle cross-sectional area in patients with COPD that relates to strength |
| Ikezoe et al., 2012 (61) | 16 young (mean age 20.3 years) and 34 elderly (mean age 84.2 years) women | - Myotonometer: muscle stiffness of quadriceps femoris muscle - Ultrasound: thickness of the rectus femoris and the vastus intermedius muscles and the overlying subcutaneous fat | Muscle stiffness during contraction, rate of change in muscle stiffness during contraction, and muscle thickness were significantly larger in young than in elderly subjects | increase in muscle stiffness during voluntary muscle contraction is limited in elderly women |
| Thomaes et al., 2012 (46) | 45 older coronary artery disease patients | - Muscle ultrasound - Computed tomography (CT) | intraclass correlation coefficient (ICC) of 0.97 for test-retest reliability of ultrasound  ICC between ultrasound and CT of 0.92  Muscle strength parameters significantly correlated with muscle diameter (range r = 0.45-r = 0.61, p < 0.05). | Ultrasound imaging as a valid and reliable measurement tool to assess the rectus femoris muscle diameter in older coronary artery disease patients |
| Cadore et al., 2012 (71) | 31 healthy elderly men (65.5 ± 5.0 years) | - Muscle ultrasound: echo intensity of rectus femoris and quadriceps femoris muscle thicknesses - Lower-body isometric and isokinetic peak torques, rate of force development | significant negative correlations between the individual values of echo intensity with the corresponding individual values of isometric and isokinetic peak torques (r=-0.48 to r=-0.64; p<0.05) | echo intensity analysis using computer-aided gray-scale analysis may contribute to the research of neuromuscular performances in the elderly |
| Fukumoto et al., 2012 (72) | 92 healthy women (70.4 ± 5.5 years) | - Muscle ultrasound: muscle thickness, subcutaneous fat thickness (FT), and echo intensity (EI) of the quadriceps femoris - Knee extensor isometric strength | EI significantly correlated with quadriceps strength independently of age or muscle thickness  Muscle thickness and EI were independently associated with quadriceps strength  no significant correlations of EI with FT | ultrasound muscle quantity and quality parameters independently contribute to muscle strength in middle-aged and elderly persons. |
| Strasser et al., 2013 (54) | 26 young (24.2 ± 3.7 years) and 26 old (age 67.8 ± 4.8 years) patients | - Muscle ultrasound: muscle thickness, pennation angle and echogenicity of quadriceps muscles - Isometric maximum voluntary contraction force (MVC) | measurements of thickness (intraclass correlation coefficients, 85-97%) had highest reproducibility.  highly significant correlation of quadriceps muscle thickness with MVC in the elderly and in the young. thickness of musculus vastus medialis had best correlation with MVC in the elderly. | ultrasound measurement of muscle thickness, especially of musculus vastus medialis, is a reliable, bedside method for monitoring the extent of sarcopenia |
| Watanabe et al., 2013 (73) | 184 elderly men (65-91 years) | - Muscle ultrasound: echo intensity (EI), muscle thickness (MT), and subcutaneous fat thickness (FT) of the anterior compartment of the right thigh - Maximum isometric torque of knee extension | significant negative correlation of EI with muscle strength (r = -0.333, P < 0.001)  MT and EI of the knee extensor muscle independently associated with maximum isometric knee extension strength | aging-related changes in muscle quality contribute to diminishing muscle strength |
| Abe et al., 2014 (45) | 41 men and 40 women aged 50 to 74 years | - Muscle ultrasound: muscle thickness (MTH) at two sites on the anterior (A50) and posterior (P50) aspects of the mid-thigh. A50:P50 MTH ratio was calculated to evaluate site-specific thigh muscle loss - Dual-energy X-ray absorptiometry (DXA) - Zigzag walking time | Anterior and posterior thigh MTH as well as A50:P50 MTH ratio was higher in men than in women.  A50:P50 MTH ratio was inversely (p < 0.05) correlated to zigzag walking time in both sexes | measurement of thigh MTH ratio using ultrasound and zigzag walking performance may be possible criteria for assessing site-specific thigh sarcopenia in middle-aged and older men and women |
| Scanlon et al., 2014 (67) | 25 older adults | - Dual-energy X-ray absorptiometry (DXA) - Muscle ultrasound - muscle strength, quality, and architecture by knee extension | Resistance training increases CSA of the vastus lateralis increased by 7.4% (p ≤ 0.05).  Physiological cross-sectional area of the thigh (composite measure of muscle architecture) was related significantly to strength (r = 0.57; p ≤ 0.01) | 6 weeks of resistance training increased strength, muscle quality, and muscle morphology in older adults |
| Rech et al., 2014 (69) | 45 healthy, active elderly women (70.28 ± 6.2) | - Muscle ultrasound: Quadriceps femoris muscle thickness and echo intensity (EI) - Maximal isometric voluntary contraction curves | EI of quadriceps femoris muscles correlated negatively with several functional tests such as 30-s sit-to-stand-up (30SS) test, usual gait speed (UGS) test, and isometric peak torque | muscle echointensity may be an important predictor of functional performance and knee extensor power capacity in elderly, active women |
| Abe et al., 2014 (90) | 1994 Japanese nonobese men and women aged 20 to 85 years | - Muscle ultrasound: muscle thickness at six sites on the anterior and posterior aspects of the body - Site-specific thigh sarcopenia was calculated using muscle thickness at the anterior and posterior aspects of the thigh | Age was inversely correlated to total skeleton muscle mass index and site-specific thigh sarcopenia in men (r=−0.480 and r=−0.522) and women (r=−0.243 and r=−0.516). Compared to the sarcopenia estimated by total skeleton muscle mass index, there was a higher prevalence of site-specific thigh sarcopenia observed in both sexes. | site-specific thigh sarcopenia appears before it is able to be detected at the whole body level |
| Ismail et al., 2015 (43) | 20 community-dwelling female subjects | - Dual energy X-ray absorptiometry (DXA) - Muscle ultrasound | 10 women with low LBM (lean body mass) had higher scaled peak force, lower BMI, and lower echogenicity values in comparison to 10 women with normal LBM (p < 0.05). | ultrasound morphometry values are associated with LBM |
| Berger et al., 2015 (44) | 54 adults (20-55 years) and 51 adults > 60 years | - Muscle ultrasound of rectus femoris muscle - Dual energy X-ray absorptiometry (DXA) - Quadriceps isometric strength - 12 minutes' walk | ultrasound measurements of the rectus femoris miscle correlated significantly with lean body mass estimated by DXA | ultrasound of rectus femoris may be a reliable and accurate method to evaluate muscle mass in older people |
| Minetto et al., 2016 (93) | 30 women and 14 men (67-93 years) and 60 younger individuals (30 women and 30 men, 20-36 years) | - Bioelectrical impedance analysis (BIA) - Muscle ultrasound: body composition and thickness of rectus femoris, vastus lateralis, tibialis anterior, medial gastrocnemius | site-specific cut-points for muscle thickness:  rectus femoris: 20 mm in men and 16 mm in women;  vastus lateralis: 17 mm in men and 15 mm in women;  tibialis anterior: 23 mm in men and 22 mm in women;  medial gastrocnemius: 13 mm in both men and women | muscle ultrasound provides a practical and accurate tool for identifying individuals with low muscle mass |
| Paris et al., 2017 (41) | 96 university and community dwelling adults | - Ultrasound: muscle thickness - Whole-body dual-energy X-ray absorptiometry scans: appendicular lean tissue. | The optimized protocol demonstrated a strong ability to identify low lean tissue mass (AUC 0.89). | The four-site protocol can be improved with the addition of the anterior upper arm muscle thickness, sex, and age when predicting appendicular lean tissue mass. |
| Yamada et al., 2017 (42) | 100 community-dwelling older men (81.6 ± 7.4 years) and 247 women (79.7 ± 6.9 years) | - Muscle ultrasound - Multifrequency bioelectrical impedance analysis (BIA) - Physical assessment batteries | AUC of the rectus femoris muscle thickness: 0.70 in men and 00.63 in women,  best cutoff of rectus femoris muscle thickness: 1.34 cm in men and 1.18 cm in women. AUC of echo intensity in quadriceps femoris muscle 0.66 in men and women  best cutoff of the echo intensity in the quadriceps femoris: 41.7 in men and 44.8 in women to discriminate low from normal muscle function | potential muscle quality and quantity indicators for sarcopenia diagnosis by ultrasonography and multifrequency BIA |
| Akazawa et al., 2017 (74) | 25 older women unable to walk, 22 frail older women, and 22 healthy older women | - Muscle ultrasound: quadriceps echo intensity - Quadriceps muscle strength - Gait scores | significantly negative correlation between Echo intensity (EI) in the dependent group and muscle strength (-0.635) and the functional independence measure gait score (-0.344)  EI in the dependent group significantly higher than in the healthy group  no significant difference in EI between the dependent and frail groups. | negative relationships between intramuscular fat and muscle strength, and intramuscular fat and degree of gait independence in dependent older women. |
| Akima et al., 2017 (76) | 64 (62-88 years) healthy men (n=27) and women (n=37) | - Muscle ultrasound: echo intensity and muscle thickness of the quadriceps femoris muscles - Sit-up, supine-up, sit-to-stand, 5-m maximal walk and 6-min walk tests | Quadriceps femoris echo intensity significantly correlated with muscle thickness  Stepwise multiple regression analysis with echo intensity as a dependent variable revealed muscle thickness, sit-to-stand test in men and age, and muscle thickness and sit-to-stand test in women, to be significant variables | Quadriceps femoris echo intensity can be explained by muscle thickness, sit-to-stand and/or age in older men and women |
| Kawai et al., 2018 (75) | 1239 community-dwelling older adults. | - Muscle ultrasound: subcutaneous fat thickness (FT), quadriceps muscle thickness (MT), subcutaneous fat echo intensity (FEI), and muscle echo intensity (EI) - Knee extension strength | Morphological and qualitative characteristics were classified into: (A) normal, (B) sarcopenic obesity, (C) obesity, and (D) sarcopenia type.  correlation between the ultrasound measures and knee extension strength differed among the classification types. classification types were significantly associated with sarcopenia prevalence | Classification of sonographic morphological and qualitative characteristics may be useful for assessing sarcopenia in community-dwelling older adults |
| Hida et al., 2018 (102) | 201 participants (99 male and 102 female, mean age: 66.2 years) | - Muscle ultrasound: thigh muscle thickness (TMT, sum of the rectus femoris and vastus intermedius muscle thickness) - Bioelectrical impedance analysis (BIA): appendicular skeletal muscle mass (aSMI) | TMT significantly reduced in subject with sarcopenia  Muscle measurements obtained using the BIA methods (aSMI) and ultrasound methods (TMT) showed a significant correlation, with a correlation coefficient of 0.38 (p < 0.001)  Cutoff value, sensitivity, and specificity of TMT in diagnosis of muscle loss were 36 mm, 72.0%, and 73.9%, respectively, for the male participants, and 34 mm, 72.2%, and 72.4%, respectively, for the female participants. | Ultrasound for thigh muscle might be a simple diagnostic method for sarcopenia detection |
| Alfuraih et al., 2019 (63) | 26 young (20–35 years), 21 middle-aged (40–55 years), and 30 elderly (77–94 years) volunteers | - Ultrasound: Shear wave velocity (SWV) measured in the quadriceps, hamstrings and biceps brachii | The overall resting muscle SWV gradually decreased with age but was only significantly reduced in the elderly group (p < 0.001); SWV were significantly different (p < 0.05) between young (1.77 m/s), middle-aged (1.64 m/s) and elderly (1.48 m/s). 16.5% lower muscle stiffness in the elderly group compared to the young. | decline in skeletal muscle stiffness correlating with muscle weakness in ageing |
| Ata et al., 2019 (88) | 145 healthy subjects | - Muscle ultrasound; muscle thickness, fascicule length, pennation angle - Body composition by anthropometric methods - Bioelectrical impedance analysis Functional status: hand grip strength and gait speed | Abdominal and thigh muscles were thinner and triceps muscle was thicker in older subjects  Gait speed, grip strength and regional muscle measurements decreased with age at higher rates (26-28%), skeletal muscle mass index was affected at a lower rate (15%). | Low muscle strength and regional muscle measurements should be used to confirm the diagnosis of sarcopenia |
| Rustani et al., 2019 (100) | 119 patients (average age 82 years, 50.4% females) | - Muscle ultrasound: Rectus femoris muscle thickness (in cm) | Average thickness of rectus femoris muscle was 0.78 ± 0.26  significantly lower in sarcopenic patients (0.55 ± 0.2 vs. 0.9 ± 0.3; p < 0.001) and females (0.7 ± 0.3 vs 0.86 ± 0.3; p < 0.001)  cut-off points: 0.7 cm for females, 0.9 cm for males. sensitivity of ultrasound measurement 100%, specificity 64%, positive predictive value 64.3% and negative predictive value 100% | a screening test for sarcopenia based on the ultrasound measurement of rectus femoris muscle thickness |
| Kara et al., 2020 (89) | 326 community-dwelling adults | - Bioelectrical impedance analysis: total skeletal muscle mass - Muscle ultrasound: nine-site muscle thickness - Isometric handgrip and knee extension strengths - Physical performance by usual Gait Speed, Chair Stand Test, and Timed Up and Go Test. | sonographic thigh adjustment ratio: quotient of thigh muscle thickness and body mass index  cutoff values: 1.4 for males and 1.0 for females  Sonographic thigh adjustment ratio values were negatively correlated with Chair Stand Test and Timed Up and Go Test in both sexes (all p < 0.05) and positively correlated with gait speed in female subjects and knee extension strength in male subjects (both p < 0.05). | Regional (rather than total) muscle mass measurements should be taken into consideration for the diagnosis of sarcopenia. |
| Yuguchi et al., 2020 (101) | 195 healthy Japanese aged ≥65 years (72.4 ± 4.3 years; 72 males) | - Bioelectrical impedance analysis (BIA): skeletal muscle mass index (SMI) - Muscle ultrasound: gastrocnemius thickness and collected echo intensity (CEI) | gastrocnemius thickness was lower (11.0 vs 13.3 mm; p < 0.01), and CEI higher (97.5 vs 87.0; p = 0.02) in the low SMI group than those in the normal group  cut-off value of gastrocnemius thickness: <11.6 mm (AUC, 0.83; sensitivity, 0.83; specificity, 0.73; p < 0.01). | Gastrocnemius thickness was associated with low skeletal muscle mass |
| Madden et al., 2021 (53) | 66 women and 84 men (≥ 65 years; 80.0 ± 0.5 years) | - Muscle ultrasound: vastus medialis muscle thickness - Bioimpedance assay: lean body mass (LBM) - grip strength, mid-arm biceps circumference, gait speed | vastus medialis muscle thickness significantly correlated with LBM (Standardised β = 0.204 ± 0.058, R2 = 0.577, p < 0.001) and mid-arm biceps circumference (Standardised β = 0.141 ± 0.067, R2 = 0.417, p = 0.038)  significant correlation with grip strength (Standardised β = 0.118 ± 0.115, R2 = 0.511, p < 0.001) | Point of care ultrasound shows as a potential screening tool for sarcopenia in older adults |
| Kelp et al., 2021 (82) | 15 younger (21 ± 2 years) and 15 older (70 ± 3 years) participants | - Muscle ultrasound: fascicle length, pennation angle and muscle thickness in medial gastrocnemius (MG) and lateral gastrocnemius (LG) - performed contractions at 20%, 40%, 60%, 80%, and 100% of maximum voluntary contraction (MVC) | MG and LG fascicle shortening (p < 0.001) and rotation (p < 0.001) increased with increasing contraction  Muscle thickness increased at higher contraction levels in LG, and not MG.  Increased changes in pennation angle were associated with increased muscle thickness in LG, but not MG at 80% and 100% MVC | Gastrocnemii shape changes are similar in active older and younger adults  Relationship between pennation angle and muscle thickness can differ between synergistics and across contraction levels |
| Matsuzawa et al., 2021 (98) | 58 hemodialysis patients | - Bioelectrical impedance analysis (BIA): muscle mass - Muscle ultrasound: cross-sectional area (CSA) of the rectus femoris - Muscle strength and physical performance | Ultrasound-derived muscle mass was strongly correlated with BIA-derived measurements.  Of the patients who were diagnosed with sarcopenia by the ultrasound-based criteria, 96% met the BIA-based criteria. | Ultrasound identified the patients at higher risk of skeletal muscle loss and sarcopenia with good discriminatory power |
| Tada et al., 2021 (99) | 84 patients with rheumatoid arthritis | - Muscle ultrasound: anterior thigh muscle thickness (MT) and fat thickness (FT). - Muscle and body fat mass examined by a body composition analyzer | MT was significantly lower in RA patients with sarcopenia than in those without (23.8 vs 28.2 mm, p = 0.001)  MT was related to sarcopenia (men: r = 0.56, p = 0.02, women: r = 0.32, p = 0.01)  cut-off value of MT for sarcopenia: 24.7 mm in men and 19.7 mm in women | ultrasound of the anterior thigh was useful to detect sarcopenia and obesity in RA patients. |
| Sengul Aycicek et al., 2021 (103) | 136 patients admitted to geriatrics outpatient clinic for comprehensive geriatric assessment | - Bioelectrical impedance analysis (BIA): skeletal muscle mass ındex (SMI) - Muscle ultrasound: thickness of the gastrocnemius muscle - Diagnosis of sarcopenia was made according to the EWSGOP 2 diagnostic criteria. | cut-off value of gastrocnemius muscle thickness to predict sarcopenia: ≤12.3 mm in women (AUC: 0.862,p <0.001) and ≤12.3 mm in men (AUC:0.900, p < 0.001) | Gastrocnemius thickness seems to predict low hand grip strength better than SMI measured by BIA. |
| Liu et al., 2022 (81) | 413 older adults from 9 nursing homes | - Muscle ultrasound: thickness and echo intensity of masseter muscles - Masticatory function (color-changing chewing gum), number of molar occlusal supports, comorbidity, oral diadochokinesis, maximum tongue pressure - Calf circumference | 179 (43.3%) had masticatory dysfunction  thickness of the masseter muscle and oral diadochokinesis decreased with age  echo intensity of the masseter muscle increased | dynamic change in the thickness of the masseter muscle between contraction and relaxation was positively correlated with masticatory function |
| Ozturk et al., 2022 (104) | 313 geriatric outpatients ≥65 years | - Comprehensive geriatric assessment, anthropometric measurement and handgrip strength (HGS) - Bioelectrical impedance analysis - Muscle ultrasound: Gastrocnemius medialis (GC), rectus femoris (RF), and rectus abdominis (RA) muscle thickness, RF cross-sectional area (CSA) | All muscle ultrasound parameters had positive correlations with HGS and the fat-free mass index.  inverse correlations between all muscle ultrasound parameters and the five-item sarcopenia questionnaire  Values of GC, RF, and RA muscle thickness and the RF CSA to predict sarcopenia for women/men were 13.9/13.8 mm (area under the curve [AUC]: 0.817/0.707 mm), 13/15.5 mm (AUC: 0.760/0.736 mm), 4.3/5.2 cm^2^ (AUC: 0.766/0.773 cm^2^), and 6.6/7.0 mm (AUC: 0.740/0.688 mm), respectively. | GC, RF, and RA muscle thickness and the RF CSA all may predict sarcopenia accurately in geriatric outpatients |

**Abbreviations are:** 30SS = 30-s sit-to-stand-up test, aSMI = appendicular skeletal muscle mass, AUC = area under the curve, BIA = Bioelectrical impedance analysis, CEI = collected echo intensity, COPD = chronic obstructive pulmonary disease, CSA = cross-sectional area, CT = Computerized tomography, DXA = Dual-energy X-ray absorptiometry, EI = echo intensity, EWSGOP 2 = European Working Group on Sarcopenia in Older People, FFM = whole-body fat-free mass, FEI = fat echo intensity, FT = subcutaneous fat thickness, GC = gastrocnemius medialis, HGS= handgrip strength, ICC = intraclass correlation coefficient, LBM = lean body mass, Lf = fascicle length, LG = lateral gastrocnemius, MG = medial gastrocnemius, MT = muscle thickness, MTH = muscle thickness, MVC = maximum voluntary contraction force, RA = rectus abdominis, RF = rectus femoris, SMI = skeletal muscle mass index, SWV = Shear wave velocity, theta = pennation angle, TMT = sum of the rectus femoris and vastus intermedius muscle thickness, UGS = usual gait speed test.
