# Supplementary material for: Uncovering sarcopenia and frailty in older adults by using muscle ultrasound—A narrative review
Source: Front Med (Lausanne). 2024 May 17;11:1333205. doi: 10.3389/fmed.2024.1333205 (PMC11140070; doi:10.3389/fmed.2024.1333205)
Supplement: Supplementary file 2 [file Table_2.DOCX]

**Supplementary Table S2.** Muscle ultrasound studies associated to frailty.

| Reference | Study population | Parameters measured | Results related to muscle ultrasound | Conclusions |
| --- | --- | --- | --- | --- |
| Shah et al, 2019 (110) | 65 adults ≥ 65 years with blunt trauma and injury severity scores<9 | - FRAIL scale - physician-based and subject/surrogate-based CFS9 - Mid-upper arm circumference (MUAC) and grip strength - Muscle ultrasound: muscle thickness (limbs and abdominal wall). | Muscle ultrasound of combined biceps and quadriceps thickness showed an AUC of 0.75 compared to the reference standard. | The CFS9 has excellent negative predictive value in ruling out frailty. Ultrasound of combined biceps and quadriceps has modest concordance as an alternative in trauma patients who cannot provide a history. |
| Salim et al., 2020 (108) | 49 patients > 64 years, referred to the acute care surgery service (76±8 years, 34% (n = 17) male) | - Muscle ultrasound: thigh muscle thickness was standardized to patient thigh length (U/S_whole/L_) - CT: skeletal muscle index (SMI) - Canadian Study of Healthy Aging Clinical Frailty Scale. | significant positive correlation between thigh U/S_whole/L_ /L and CT SMI  inverse correlation between thigh U/S_whole/L_/L and frailty score  association between U/S_whole/L_/L and postoperative major complications | U/S_whole/L_/L index can identify elderly frail patients with sarcopenia |
| Madden et al., 2021 (107) | 150 older adults ≥ 65 years (80.0±0.5 years, 66 women, 84 men) | - Muscle ultrasound: vastus medialis muscle thickness (MT) - Cardiovascular Health Study (CHS) index - Rockwood Clinical Frailty Scale | weak inverse association of MT with the CHS index (Standardized β = - 0.180 ± 0.080, R2 = 0.06, p = 0.027) and no association with the RCFS (p = 0.776). | caution must be used in trying to screen for frailty with a single ultrasonic measure. |
| Bencivenga et al., 2022 (49) | 136 patients aged ≥65 years | - Comprehensive geriatric assessment, frailty index (FI) - Muscle ultrasound: Muscle thicknesses (MT) of rectus femoris plus vastus intermedius | MT of rectus femoris plus vastus intermedius: 29.27 (23.08-35.7) mm.  At multivariable regression analysis, FI resulted significantly and independently associated with age and MT | muscle ultrasound may be an additional imaging domain in the assessment of frailty |
| Canales et al., 2022 (109) | 32 patients scheduled for major surgery (18 frail patients, 49 to 95 years, and 14 non-frail patients, 33 to 72 years)  and 20 healthy volunteers (28 to 61 years) | - Muscle ultrasound: quadriceps and rectus femoris - Computed tomography scans: psoas muscle area - Fried phenotype assessment. | quadriceps depth and psoas muscle area were able to identify frailty (AUC 0.80 and 0.88, respectively)  cross-sectional area of the rectus femoris is less promising (AUC 0.70) | Preoperative ultrasound measurements of quadriceps depth is promising in discriminating between frail and not-frail patients before surgery |
| Benton et al., 2022 (112) | 43 patients (mean age of 78.5) | - Fatigue, Resistance, Ambulation, Illness and Loss of Weight (FRAIL) scale - Muscle ultrasound: upper arm muscles, quadriceps muscles, and abdominal wall muscles thickness | Ultrasound measurements of the three muscle groups were not significantly different between frail and non-frail groups. Frail participants had greater biceps asymmetry (difference of 0.47 cm vs 0.24 cm, p < 0.01).  A predictive logistic regression model using average quadriceps thickness and biceps asymmetry was found to identify frail patients (AUC of 0.816).  Participants with subsequent falls had smaller quadriceps (1.18 cm smaller, p < 0.01).  Subsequently hospitalized patients were found to have smaller quadriceps muscles (0.54 cm smaller, p = 0.03) and abdominal wall muscles (0.25 cm smaller, p = 0.01). | Sonographic measurements of sarcopenia in older patients had mild to moderate associations with frailty, falls and subsequent hospitalizations. |
| Anderson et al., 2023 (111) | 223 adult prevalent (≥3 months) haemodialysis recipients | - Muscle ultrasound: bilateral anterior thigh thickness (BATT) - Frailty Phenotype (FP) - Frailty Index (FI) - Edmonton Frailty Scale (EFS) - Clinical Frailty Scale (CFS) | BATT was associated with increasing frailty on simple linear regression by all frailty tools, but lost significance on addition of covariables.  Upon dichotomising frailty tools into Frail/Not Frail, BATT was associated with frailty by all tools on univariable analyses, but only retained association for EFS on the fully adjusted model (OR 0.97, 95% C.I. 0.94-1.00, p = 0.05). | Ultrasound measures of quadriceps thickness is variably associated with frailty in prevalent haemodialysis recipients. |

**Abbreviations are:** AUC = area under the curve, BATT = bilateral anterior thigh thickness, CFS = Clinical Frailty Scale, CHS = Cardiovascular Health Study, C.I. = confidence interval, EFS = Edmonton Frailty Scale. FI = frailty index, FP = Frailty Phenotype, FRAIL = Fatigue, Resistance, Ambulation, Illness and Loss of Weight scale, MT = muscle thickness, MUAC= Mid-upper arm circumference, OR = odds ratio, SMI = skeletal muscle mass index.
